# Perception and Practice of Kangaroo Mother Care Among Mothers of Pre-term Babies at a National Referral Hospital in a Limited Resource Setting

**DOI:** 10.24248/eahrj.v8i1.753

**Published:** 2024-03-28

**Authors:** Beatrice Afande Mukhola, Angeline C Kirui, Lucy W Kivuti-Bitok

**Affiliations:** a Department of Nursing Sciences, University of Nairobi, Nairobi, Kenya; b Kenyatta National Hospital, Nairobi, Kenya

## Abstract

**Introduction:**

The effect of COVID-19 on KMC practices in limited resource settings and on healthcare delivery globally remains unclear.

**Methods:**

In this cross-sectional descriptive study, we aimed to assess the perceptions and practice of Kangaroo mother care (KMC) during the COVID-19 pandemic by postnatal mothers of preterm babies in the New Born Unit (NBU) at Kenyatta National Hospital.

**Results:**

A total of 82 postnatal mothers of preterm babies participated in this study. Majority of respondents practiced KMC during the pandemic period, with intermittent KMC being the most common form of practice. The reasons for practicing KMC were to promote mother-baby bonding and facilitate infant breastfeeding, while the main reason for fear of practicing KMC was concerns that the baby could contract COVID-19. We found no significant associations between KMC practice and education level, religion, pre-existing illness, and knowledge about COVID-19. It was noted that older mothers and those with more children were less likely to engage in KMC. There was a trend for married women to be more likely to engage in KMC, even though this did not reach statistical significance.

**Conclusion:**

There is a need to reinforce continued KMC practice during the pandemics and allay any concerns that mothers have over practicing KMC. We emphasise the need to prioritise KMC practices in the context of possible future pandemics, as it has been shown to have numerous benefits for preterm babies and their mothers.

## INTRODUCTION

Kangaroo mother care (KMC) is a method of care for newborns that involves early, continuous, and prolonged skin-to-skin contact (SSC) between the mother and preterm babies. It is often carried out concurrently with breastfeeding at the hospital and may be continued after discharge.^1^ UNICEF has identified KMC as a universally available and biologically sound method of care for all newborns, particularly premature and low birthweight infants, in both developed and developing countries, as stated by Moore.^2,3^

KMC promotes parent-child attachment, improves parental confidence as caregivers, and promotes infant nutrition.^2^ Preterm infants receiving KMC experience more normalised vital signs, increased weight gain, and fewer nosocomial infections.^4–6^ Additionally, KMC has been shown to improve cognitive development, normalise growth, reduce pain responses, have positive effects on motor development, improve sleep patterns, and ameliorate colic colic.^7,8^

The benefits to health institutions using KMC include reduced need for the more expensive conventional neonatal care of preterm babies and increased parental involvement and opportunities for health education.^9^ In general, there is evidence that KMC, when compared to conventional neonatal care in resource-limited settings, significantly reduces the risk of mortality in clinically stable premature infants.^10^

KMC has been threatened by the COVID-19 pandemic, which has caused worldwide concern and has become a threat to global public health.^11,12^ The pandemic triggered a crisis in global health systems, severely undermining the effective delivery of health care services throughout the world.^13^ The additional burden of planning and responding to the COVID-19 pandemic runs the risk of overwhelming health systems, leaving ongoing preventive care, such as maternal and child health services, severely disrupted.^14^ The effect of Covid-19 pandemic on KMC practices in limited resource setting and on health care services delivery across the globe remains unclear.^15^

The COVID-19 pandemic may have further exacerbated health system challenges and hindered the implementation of KMC in developing countries. It has been reported that the COVID-19 pandemic has resulted in a decline in the utilisation of KMC in India as resources and personnel have been redirected towards pandemic response efforts.^15^ Additionally, the fear of infection among mothers and healthcare providers has been reported to contribute to a reduction in the number of hospital visits, which has negatively impacted the initiation and continuation of KMC.^16^

Implementation of KMC practices in limited resource settings could have a significant impact on reducing neonatal morbidity and mortality rates, especially in the context of the pandemic where access to hospital care may be limited. At the same time, WHO acknowledges that it is important for governments and their partners across the world to ensure critical maternal and child health care services continue to receive the attention they require.^17^

It is therefore important to understand the challenges and opportunities faced by carers in implementing KMC during these times. We sought to assess the perceptions and practice of KMC during the COVID-19 pandemic by postnatal mothers of preterm babies in the Newborn Unit at Kenyatta National Hospital (KNH), add to the growing body of literature on the impact of the COVID-19 pandemic on KMC, and implore preparedness on lessons learnt for possible future pandemics.

## METHODS

### Study Design

A hospital-based, descriptive cross-sectional study that assessed the implementation of kangaroo mother care during the COVID-19 pandemic and identified perceptions that could be both facilitators and barriers to the practice.

### Study Area

The study was conducted at Kenyatta National Hospital's (KNH) Newborn Unit (NBU). The hospital is located in the city of Nairobi, Kenya. KNH is one of the largest public referral hospitals in the country, with a bed capacity of approximately 2,500. It offers a wide range of specialised in-patient and out-patient health care services, including radiotherapy, heart surgery, neurosurgery, oncology, diabetic care, renal dialysis and kidney transplant operations, plastic and reconstructive surgery, orthopaedic surgery, and burn management. This study focused on the practice of KMC in the NBU, which has a single room with a bed capacity of 10 for continuous KMC.

Continuous KMC entails the mother having skin-to-skin contact with the baby throughout, save when she is on a bathroom break or when absolutely necessary. Prior to the COVID-19 pandemic, the mothers would get help from their significant other during such eventualities. However, KNH had a no-visit policy during the COVID-19 pandemic, and this may have affected continuous KMC. On the other hand, mothers practice intermittent KMC for approximately 30 to 40 minutes, usually every 3 hours during feeding time. On average, KNH handles approximately 120 cases of premature infants in its NBU per month. In addition to its healthcare services, the hospital also facilitates medical training and research, making it an ideal setting for our study. By conducting our research in this environment, we were able to gather valuable insights into the implementation of kangaroo mother care in a busy, resource-limited public hospital.

### Study Population

The study population was comprised of postnatal mothers of preterm babies who were admitted to KNH's NBU during the study period. The study recruited all eligible mothers in the NBU who consented to participate in the study.

Mothers who were either critically ill or had critically ill preterm babies were excluded from the study. A critically ill mother was operationally defined as any mother who was not well enough to move from the postnatal ward to the newborn unit. We made this decision because critically ill mothers may not have been able to provide accurate information about their experiences with KMC, which could have compromised the quality of our findings. By focusing on postnatal mothers of preterm infants in the NBU, we aimed to gather first-hand perspectives on the implementation of kangaroo mother care and identify potential barriers and enablers to the practice in a real-world clinical setting.

### Sample size determination

The study sample size, for the postnatal mothers of preterm babies, was calculated using the Fishers et al^18^ formula at a 95% confidence interval, resulting in 91 postnatal mothers of preterm babies at the NBU of KNH.

### Sampling Procedure

Using the average number of premature birth cases recorded weekly in KNH in 2019, which was 30, and the desired sample size of 91, the sampling interval (k) was calculated as follows:

k = n/s = 91/30 = 3.03

Where n is the sample size and s is the average number of preterm birth cases recorded weekly in KNH in 2019.

Consequently, every third mother of a preterm baby admitted to KNH's Newborn Unit was recruited into the study until the desired sample size was achieved. This systematic sampling approach allowed us to select a representative sample of mothers who had experienced kangaroo mother care in the NBU and provided us with a diverse range of perspectives and experiences.

### Ethical Considerations

Permission to conduct the study was granted by the KNH-UoN Ethics and Review Committee. (Approval number: P372/05/2021, received from KNH/UoN ERC). We also obtained permission from the administration of KNH's paediatrics department to conduct the study at the NBU. Written informed consent was obtained from all participants after explaining to them the study's objectives, procedures, and potential risks and benefits. Participants were informed that their participation was entirely voluntary and that they could withdraw from the study at any time without fear of reprisal. COVID-19 prevention protocols were observed during data collection to ensure the safety of both the study's participants and the research team. The study was conducted in a manner that was respectful, transparent, and safe for all involved.

### Data Collection and Management

Several measures were taken to ensure effective data collection and management throughout the study. The mothers and research assistants sat in a private room and maintained social distancing as well as observing other COVID-19 precautionary measures. Data was collected using a researcher-administered questionnaire that was designed specifically for this study. The data collection involved the investigator asking the participants the questions contained in the research tools and documenting their responses. The respondents were given sufficient time to answer the questions, as contained in the study tools, without the research assistant interfering with their responses. Once the study participants responded to the research instruments, the research assistant reviewed the questionnaire for completeness. The questionnaire consisted of several sections that covered respondents' demographic characteristics, their perceptions, and their practice of KMC during the COVID-19 pandemic.

The study tool was pretested at a county referral facility to test its effectiveness. This allowed us to identify any areas of the questionnaire that needed to be revised or clarified before administering it to the study's participants.

After data collection, the data was fed into SPSS version 24 to quantitatively analyse the data. Descriptive statistics were employed to summarise the data, and the results were presented as percentages and frequencies. The hypothesis was tested using Chi-square tests at a significance level of *p* <.05. To protect the confidentiality and anonymity of our study participants, data was stored in a secure location, and unique identification codes were used to label each participant's questionnaire. Only members of the research team had access to the data, and all personal identifying information was destroyed after the completion of the data analysis process.

## RESULTS

### Sociodemographic Characteristics of the Respondents

Out of the targeted 91 postnatal mothers of preterm babies, we were able to obtain adequate responses from 82 of the mothers, translating into a response rate of 90.1%. The majority of the mothers (47.6%) were aged between 30 and 39 years and had a secondary level of education (48.8%). Most (76.8%) of the respondents were married and had more than one child (54.9%). The majority of the respondents were Christians (92.7%) and did not have any pre-existing illnesses (76.8%). Nearly all of the respondents had heard about COVID-19 (98.8%), correctly identified how COVID-19 was transmitted (89.0%), and identified the symptoms of COVID-19 infection (95.1%). The findings were presented in Table 1.

**TABLE 1:** Demographic Characteristics and Factors Associated with Kangaroo Mother Care Practice among Mothers of Preterm Babies at Kenyatta National Hospital during the COVID-19 Pandemic

Characteristic	Frequency	Percent	Chi-Square *(P-value)*
Age			
18 - 29 years	31	37.8	6.09 (.014)*
30 - 39 years	39	47.6	
40 - 49 years	12	14.6	
Education level			
No formal education	1	1.2	4.09 (.128)
Primary	9	11.0	
Secondary	40	48.8	
Tertiary	32	39.0	
Marital status			
Single	15	18.3	3.43 (.064)
Married	63	76.8	
Separated	2	2.4	
Divorced	1	1.2	
Widowed	1	1.2	
Number of children			
One child	37	45.1	10.39 (.001) **
2 - 3 children	37	45.1	
4 - 6 children	8	9.8	
Religion			
Christian	76	92.7	0.17 (.682)
Muslim	6	7.3	
Any pre-existing illness			
Yes	19	23.2	0.15 (.698)
No	63	76.8	
Heard of KMC?			
Yes	75	91.5	0.001 (.974)
No	7	8.5	
Heard about Covid-19			
Yes	81	98.8	0.03 (.862)
No	1	1.2	
Correctly identified how Covid-19 was transmitted (*n*=81)			
Yes	73	89.0	0.63 (.427)
No	9		
Correctly identified the symptoms of Covid-19 infection (*n*=81)			
Yes	77	95.1	3.69 (.055)
No	4	4.9	
Mother Practicing KMC During Covid-19 pandemic period			
Yes	62	75.6	
No	20	22.4	
Form of KMC they were practicing			
Continuous	12	19.4	
Intermittent	50	80.6	

### Kangaroo Mother Care Practice during the Covid-19 Pandemic

During the COVID-19 pandemic period, 75.6% of mothers practiced KMC, with intermittent KMC being the most common form of practice (80.6%). The chi-square test results showed that age (*P.014*), the number of children *(P.001*), and possibly marital status (*P.064*) were associated with KMC practice, whereas education level, religion, pre-existing illness, and knowledge about COVID-19 were not significantly associated with KMC practice. It was noted that older mothers and those with more children were less likely to engage in KMC. There was a trend for married women to be more likely to engage in KMC, even though this did not reach statistical significance.

### Reasons for and against Kangaroo Mother Care during the Covid-19 Pandemic: Perceptions and Concerns of Mothers of Preterm Babies.

Mothers reported several reasons for support and concerns about practicing KMC during the COVID-19 pandemic. Table 2 displays the reasons reported by postnatal mothers of preterm babies for practicing continuous KMC during the COVID-19 pandemic, as well as the factors that contributed to their fear of practicing KMC. The most common reasons for practicing KMC despite the pandemic were promoting mother-baby bonding (40.3%) and facilitating infant breastfeeding (25.8%). Other reasons included keeping the infant warm (14.5%), aiding in infant weight gain (9.7%), and improving infant sleep patterns (6.5%).

**TABLE 2:** Summary of Mothers' Reasons for Practicing Continuous KMC, Fears, and Concerns about KMC during the COVID-19 Pandemic in the Newborn Unit of KNH

Reasons Identified for Practicing Continuous Form of KMC Even in the Face of COVID-19 Pandemic	Frequency	Percent
It promoted mother-baby bonding	25	40.3
It promoted infant breastfeeding	16	25.8
It helped keep the infant warm	9	14.5
It enhanced mother's confidence in infant caregiving	2	3.2
It helped infants gain weight	6	9.7
It improved infant's sleep patterns	4	6.5
Reasons identified of being scared of practicing KMC during COVID-19 pandemic		
Perceiving the baby as being too vulnerable	9	19.1
Fears that they too could contract COVID-19 infection	7	14.9
Low awareness as to how COVID-19 could affect the baby	4	8.5
Lack of guidance as to how to perform KMC in light of COVID-19	2	4.3
General fear over the health and wellbeing of the baby in the face of the COVID-19 pandemic	6	12.8
Mothers' Concerns over KMC Practice during the COVID-19 Pandemic		
Fear over the baby contracting COVID-19	70	85.4
Fear of coming into contact with contaminated services	14	17.1
Fear that they could contract COVID-19 and pass the same to the infant	42	51.2
Not knowing the COVID-19 status of the healthcare providers who interacted with the infants	8	9.8
Lack of awareness about the potential effects of COVID-19 on the infants	27	32.9

The most common reasons for fear of practicing KMC during the pandemic were concerns that the baby could contract COVID-19 (85.4%). Additional reasons included fear of contracting COVID-19 and transmitting it to the infant (51.2%), lack of awareness about the potential effects of COVID-19 on infants (32.9%), apprehension about coming into contact with contaminated surfaces (17.1%), and uncertainty about the COVID-19 status of healthcare providers who interacted with the infants (9.8%).

## DISCUSSION

Our aim was to establish the perception and practice of KMC among mothers of preterm babies at the newborn unit of KNH and establish associated factors.

### Kangaroo Mother Care Practice during the Covid-19 Pandemic

We established that most of the mothers of preterm babies at KNH were aware of KMC and understood it as skin-to-skin contact between the mother and the baby. The mothers were of the view that KMC was important as it promoted bonding between the mother and the baby, helped keep the baby warm, promoted breastfeeding, enhanced mothers' caregiving experience, helped infants gain weight, and improved their sleeping patterns. Studies by Boundy et al^2^ and Norén et al^5^ reported similar findings where participating mothers were fairly knowledgeable about KMC, particularly regarding what KMC was and its benefits to the mother and the infant. A similar observation on the positive impact of an empowered mother was also made by Abdul-Mumin et al^19^. Conversely, studies by Uwaezuoke^4^ and a review by Chan et al^8^ noted gaps in postnatal mothers' awareness of kangaroo mother care, especially in terms of why it was a critical intervention.

Mothers of preterm babies at KNH demonstrated adequate knowledge of the COVID-19 pandemic, as they were able to correctly identify the symptoms of COVID-19 and how it is transmitted. Similar results were reported in studies by Goyal et al ^20^ and Singh et al^14^, where the participants, who were postnatal mothers, were found to be adequately aware of the ongoing COVID-19 pandemic, including basic information relating to the COVID-19 infection. These sentiments were also shared by Paul et al ^21^ and Minckas et al.^22^ Conversely, low awareness about the COVID-19 pandemic was reported among postnatal mothers in studies in Mozambique by Pires et al^24^, and Hailemariam et al^25^ in Ethiopia.

During the ongoing COVID-19 pandemic, most mothers of preterm babies at KNH practiced intermittent kangaroo mother care, while a few others did not practice KMC. This was largely due to fears of COVID-19 transmission among the mothers and fear of possible transmission of the infection to their babies, especially in light of the babies' delicate health status. This contributed to the decline in the practice of KMC. WHO1 and UNICEF^26^ reported notable declines in the number of women offering the KMC intervention to their preterm infants in various settings during the COVID-19 pandemic period compared to the period before the emergence of the current pandemic. Studies by Kimani et al^27^ das Neves Pires et al^23^ and *Okereke* et al^28^ have also reported disruptions of the COVID-19 pandemic on preterm mothers' utilisation of KMC.

### Perception of Mothers of Preterm babies towards Kangaroo Mother Care Practice during the Covid-19 Pandemic

Mothers of preterm babies at KNH showed support for the practice of KMC during the COVID-19 pandemic, recognising the significant benefits it provides to their infants, as long as the recommended infection prevention measures were followed. This supports previous findings by Abdul-Mumin et al^19^ and Karkee et al^29^ who found that surveyed mothers were supportive of KMC even during the pandemic. These mothers were aware of and appreciated the benefits of KMC for their preterm infants. However, the mothers also expressed fear and concerns about the pandemic and its potential impact on their babies during KMC. They were worried about contracting COVID-19 themselves and transmitting it to their babies, perceived their babies to be especially vulnerable to the infection, and lacked guidance on how to conduct KMC safely in light of COVID-19. This observation is consistent with the findings of the study by Busch-Hallen et al^30^ who reported that although most mothers positively perceived KMC, a significant proportion of them were hesitant to practice it during the pandemic period. Stuebe^31^ also noted that some mothers expressed reservations about KMC during the pandemic, despite their positive perception of the practice. Similarly, Stuebe^31^ found that mothers' acceptance of KMC significantly decreased due to fear of COVID-19 transmission. In Ethiopia, Temesgen et al^32^ reported that some mothers were hesitant to practice KMC during the pandemic due to the risk of COVID-19 transmission.

### Study Limitations

Our study had several limitations that should be taken into consideration when interpreting the results. Firstly, the study was conducted in a single hospital, limiting the generalizability of the findings to other settings. Secondly, the cross-sectional nature of the study means that causality cannot be established between the investigated variables. Additionally, relying on self-reported data may have introduced social desirability bias and recall bias. Furthermore, the study only included mothers who were able to respond to the survey, which may have introduced selection bias. Additionally, the study did not explore the experiences and perceptions of healthcare providers regarding KMC during the Covid-19 pandemic, which could have provided valuable insights on the challenges and facilitators of KMC practice. Finally, the study did not investigate the impact of KMC practice on infant outcomes during the Covid-19 pandemic, which is an important area for future research. Despite these limitations, our study provides valuable insights into the KMC practices and perceptions of mothers of preterm babies during the pandemic in a limited-resource setting. Our results indicate that many mothers persisted in employing KMC during the pandemic, despite apprehensions about COVID-19 transmission. This underscores the necessity of furnishing mothers with precise guidance and assistance to sustain safe KMC practices amid such crises. Subsequent studies ought to explore methods for alleviating maternal concerns about KMC in potential future pandemics.

## CONCLUSION

In conclusion, while mothers of preterm babies at KNH recognise the benefits of KMC, during the COVID-19 pandemic, KMC was practiced intermittently due to concerns and fears of being infected or infecting the neonates during the KMC process. Addressing these concerns and providing guidance on safe KMC practices during the pandemic can help mothers feel more comfortable and increase the likelihood of continued KMC practices. The following recommendations are made based on these findings: i) The hospital should provide health education to mothers of preterm babies to address the fears and misconceptions associated with KMC during the pandemic; ii) Further studies should be conducted to investigate the KMC practices of mothers of preterm babies in other settings, especially in areas with limited resources, to establish the generalizability of the findings of this study; and iii) the hospital should also provide counselling services to address any challenges they may encounter during the practice.
